# Neuroprotective activation of astrocytes by methylmercury exposure in the inferior colliculus

**DOI:** 10.1038/s41598-019-50377-9

**Published:** 2019-09-25

**Authors:** Yasuhiro Ishihara, Kouichi Itoh, Ami Oguro, Yoichi Chiba, Masaki Ueno, Mayumi Tsuji, Christoph F. A. Vogel, Takeshi Yamazaki

**Affiliations:** 1 Program of Biomedical Science, Graduate School of Integrated Sciences for Life, Hiroshima University, Hiroshima, 739-8521 Japan; 2 Center for Health and the Environment, University of California, Davis, CA 95616 USA; 3 Laboratory for Pharmacotherapy and Experimental Neurology, Kagawa School of Pharmaceutical Sciences, Tokushima Bunri University, Kagawa, 769-2193 Japan; 4 Department of Pathology and Host Defense, Faculty of Medicine, Kagawa University, Kagawa, 761-0793 Japan; 5 Department of Environmental Health, University of Occupational and Environmental Health, Fukuoka, 807-8555 Japan; 6 Department of Environmental Toxicology, University of California, Davis, CA 95616 USA; 7 Program of Life and Environmental Sciences, Graduate School of Integrated Sciences for Life, Hiroshima University, Hiroshima, 739-8521 Japan

**Keywords:** Neuroscience, Health occupations

## Abstract

Methylmercury (MeHg) is well known to induce auditory disorders such as dysarthria. When we performed a global analysis on the brains of mice exposed to MeHg by magnetic resonance imaging, an increase in the T1 signal in the inferior colliculus (IC), which is localized in the auditory pathway, was observed. Therefore, the purpose of this study is to examine the pathophysiology and auditory dysfunction induced by MeHg, focusing on the IC. Measurement of the auditory brainstem response revealed increases in latency and decreases in threshold in the IC of mice exposed to MeHg for 4 weeks compared with vehicle mice. Incoordination in MeHg-exposed mice was noted after 6 weeks of exposure, indicating that IC dysfunction occurs earlier than incoordination. There was no change in the number of neurons or microglial activity, while the expression of glial fibrillary acidic protein, a marker for astrocytic activity, was elevated in the IC of MeHg-exposed mice after 4 weeks of exposure, indicating that astrogliosis occurs in the IC. Suppression of astrogliosis by treatment with fluorocitrate exacerbated the latency and threshold in the IC evaluated by the auditory brainstem response. Therefore, astrocytes in the IC are considered to play a protective role in the auditory pathway. Astrocytes exposed to MeHg increased the expression of brain-derived neurotrophic factor in the IC, suggesting that astrocytic brain-derived neurotrophic factor is a potent protectant in the IC. This study showed that astrogliosis in the IC could be an adaptive response to MeHg toxicity. The overall toxicity of MeHg might be determined on the basis of the balance between MeHg-mediated injury to neurons and protective responses from astrocytes.

## Introduction

Mercury (Hg) has always been present in the environment because of its natural deposition and is converted by microorganisms present in sediments to methylmercury (MeHg), which biomagnifies in the aquatic food chain. Humans are exposed to MeHg primarily through the consumption of contaminated fish, particularly large predatory fish species. Once MeHg passes into the blood, it can easily pass through the blood–brain barrier as a cysteine conjugate mainly using the neutral amino acid transport system^1^. Therefore, MeHg shows strong neurotoxicity. The major symptoms of MeHg intoxication are cerebellar ataxia, concentric contraction of the visual field, auditory disorder and dysarthria. In the case of Minamata disease, high concentrations of MeHg were detected in the brains of the patients, and several pathological findings, such as cell shedding from the precentral cortex and auditory cortices and abnormal astrocytic proliferation (gliosis) in the calcarine cortex and cerebellar granular layer, were detected^2,3^.

Oxidative stress is considered to contribute to neuronal injury elicted by MeHg. The mechanism by which MeHg induces reactive oxygen species (ROS) generation in neurons can be primarily due to the inhibition of antioxidant enzymes induced by MeHg. MeHg is able to directly bind to the selenocysteine residue in glutathione peroxidase (GPx) to increase the concentration of hydrogen peroxide inside cells by inhibiting its activity^4^. MeHg also inhibits the activity of glutathione reductase to attenuate the regeneration of reduced glutathione^5^. We previously reported that ROS generated inside cells by MeHg exposure are amplified in mitochondria to induce further neuronal damage^6^, suggesting that mitochondria play an important role in MeHg-induced neuronal injury. Some reports have suggested possible mechanisms of MeHg-induced neuronal damage other than oxidative stress, including disruption of microtubules^7^, decreases in intracellular ATP^8^ and apoptosis^6^. Thus, MeHg can injure neurons by multiple and complicated mechanisms.

Recently, it was demonstrated that glial cells show an adaptive response to protect neurons from MeHg damage. Astrocytic nuclear factor, erythroid 2 like 2 protects neurons from MeHg toxicity by upregulating antioxidative enzymes and metabolizing enzymes^9^. In addition, astrocytes respond to MeHg by releasing the proinflammatory cytokine Interleukin-6 (IL-6) to outside the cells. IL-6 plays a fundamental role in neuronal disorders accompanied by neuroinflammation. However, IL-6 induced by MeHg can attenuate MeHg toxicity in neurons^10^, although the mechanism is unclear. We previously reported that astrocytes exposed to MeHg produce and release nerve growth factor and brain-derived neurotrophic factor, which act on neurons to suppress MeHg injury^11^. Shinozaki *et al*. showed role of the astrocyte-microglia interaction in protecting neurons from MeHg damage^12^. Microglia can sense a low concentration of MeHg to release ATP outside of cells via a MAPK p38-dependent pathway. The astrocytic P2Y1 receptor recognizes released ATP and then the cells produce and release IL-6. These glial responses to MeHg for neuroprotection could be a series of adaptive responses to MeHg toxicity. Overall damage induced by MeHg might result from the balance between a harmful effect of MeHg on neurons and a glial adaptive response against MeHg.

One of the major symptoms of MeHg poisoning is abnormal auditory function. Patients with Minamata disease and Niigata Minamata disease show auditory disorders, and Faroese children exposed to MeHg exhibit abnormalities in brainstem auditory-evoked potential latencies^2,3,13,14^. These auditory injuries explain the damage to the auditory cortex by MeHg as well as the abnormality of auditory brainstem responses (ABRs) in the inferior colliculus (IC), a relay nucleus of the auditory pathway from the cochlea to the auditory cortex^13^. MeHg exposure induces decreases in Na^+^/K^+^-ATPase activity and increases in the production of nitric oxide, but further study is needed to reveal the event induced by MeHg in the auditory pathway^15^. We analyzed the brains of mice treated with MeHg time-dependently by magnetic resonance imaging (MRI) sequences, T1-weighted imaging (T1WI), T2-weighted imaging (T2WI) and diffusion-weighted imaging (DWI), and we found a high intensity of the T1 signal in the IC even before the lack of coordination evaluated by a rotarod test. Therefore, in this study, we examined MeHg-induced phenomena in the IC and uncovered the relationships between the event and auditory dysfunction.

## Results

### IC dysfunction in mice exposed to MeHg

**Figure 1:**
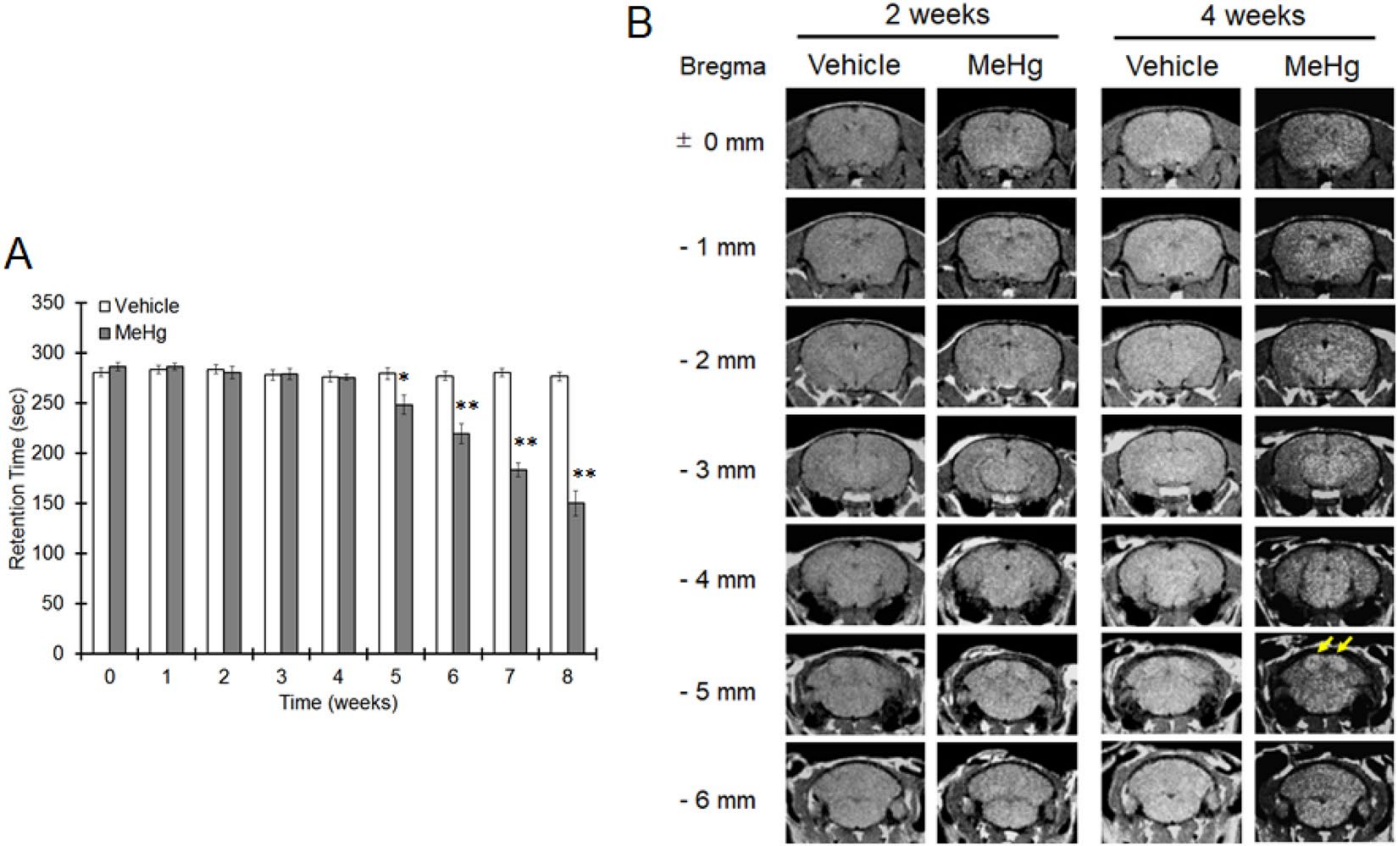
High T1 intensity detected in the IC before coordinated movement dysfunction induced by MeHg. MeHg was administered to mice at a dose of 4 mg/kg/day for 8 weeks. (**A**) Coordinated movement was measured by a rotarod test. The reported values are the mean ± S.E. (n = 10 animals in each group). Data were analyzed using a one-way ANOVA, followed by Student’s t test. *P < 0.05, **P < 0.01 vs. the vehicle group. (**B**) Representative T1-weighted images of mice treated with MeHg for 2 or 4 weeks obtained by MRI are shown. Arrows indicate the IC region.

**Figure 2:**
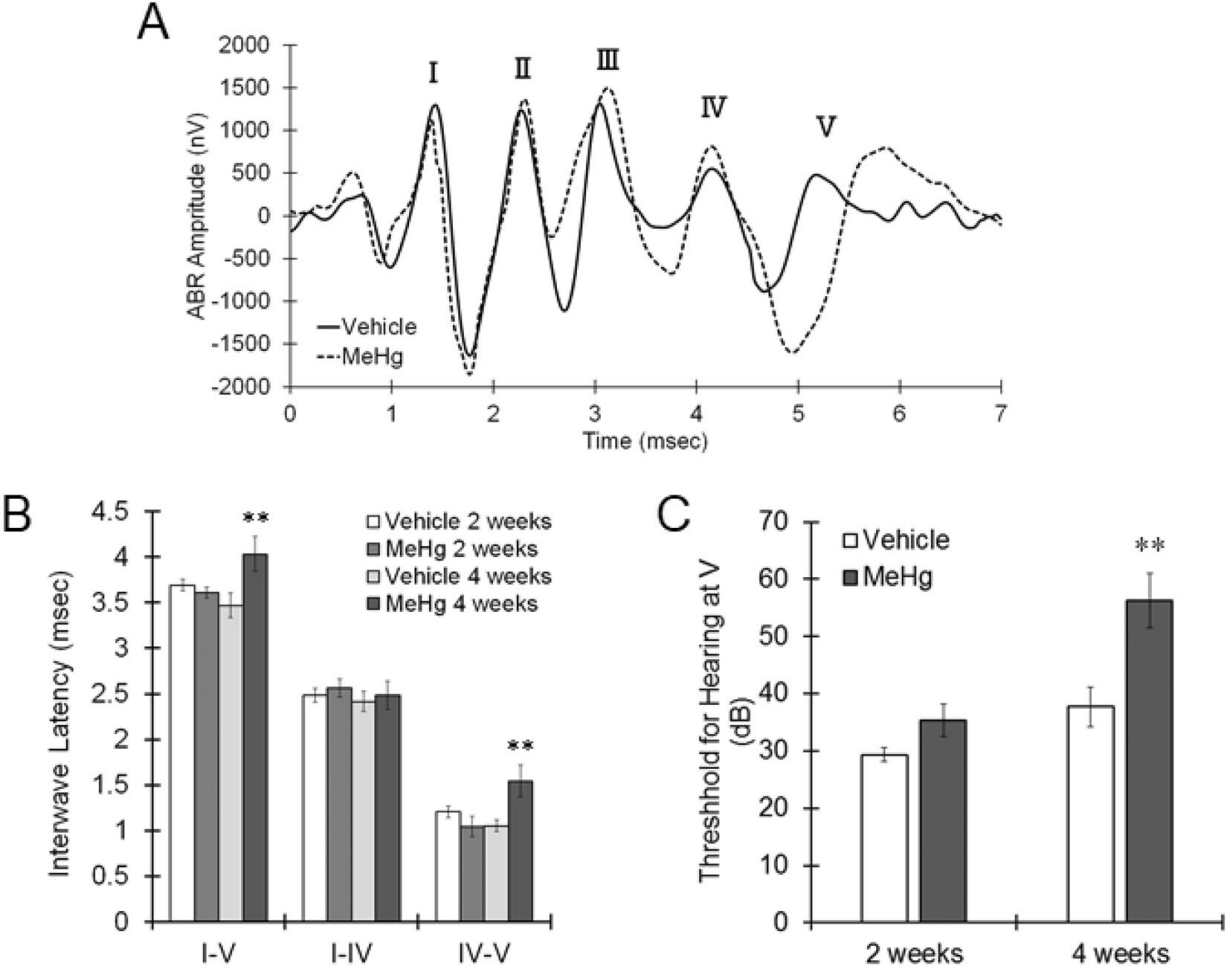
Changes in the ABR wave V latency and threshold elicited by MeHg. MeHg was administered to mice at a dose of 4 mg/kg/day for 4 weeks. Auditory function was evaluated by ABR measurement. (**A**) Representative images of the auditory potential evoked by 80 dB sound pressure in mice treated with MeHg for 4 weeks. (**B**) Interwave latency from wave I to wave V, from wave I to wave IV and from IV to V in MeHg-treated mice was measured. The reported values are the mean ± S.E. (n = 5 animals in each group). Data were analyzed using a one-way ANOVA, followed by Student’s t test. **P < 0.01 vs. vehicle group with the same age. (**C**) Mice were simulated by different sound pressures with a monitoring evoked potential at wave V to calculate the hearing threshold. Data were analyzed using a one-way ANOVA, followed by Student’s t test. **P < 0.01 vs. vehicle group with the same age.

MeHg was orally administered to male ICR mice at a dose of 4 mg/kg/day, and then coordinated movement was measured by rotor rod test once a week (Fig. 1A). MeHg exposure showed no effect on retention time on the rod 4 weeks after the beginning of administration, but after 5 weeks, the retention time on the rod decreased in a time-dependent manner compared with that for vehicle-treated mice (Fig. 1A). T1WI showed lateral ventricular enlargement and high intensity in the IC (indicated by arrows) in mice treated with MeHg for 4 weeks (Fig. 1B). There was no change other than ventricular enlargement evaluated by T2-weighted and diffusion-weighted images between vehicle- and MeHg-treated mice (data not shown). The IC is the relay nucleus of the auditory pathway from the cochleae to the primary auditory cortex, and dysfunction of the IC causes hearing loss. Thus, we next measure the function of the auditory pathway by ABR. There was no difference in the evoked potential between vehicle- and MeHg-treated mice after 2 weeks (Fig. 2B,C). However, the evoked potential at the IC was significantly delayed by 4 weeks of MeHg exposure (Fig. 2A,B). In addition, the ABR threshold at the IC significantly increased 4 weeks after MeHg treatment (Fig. 2C). These results indicate that MeHg induces IC dysfunction before coordinated movement disorders occur, although IC impairment is considered not to be involved in the dysfunction of coordinated movement.

### Astrocytic activation in the IC of mice treated with MeHg

**Figure 3:**
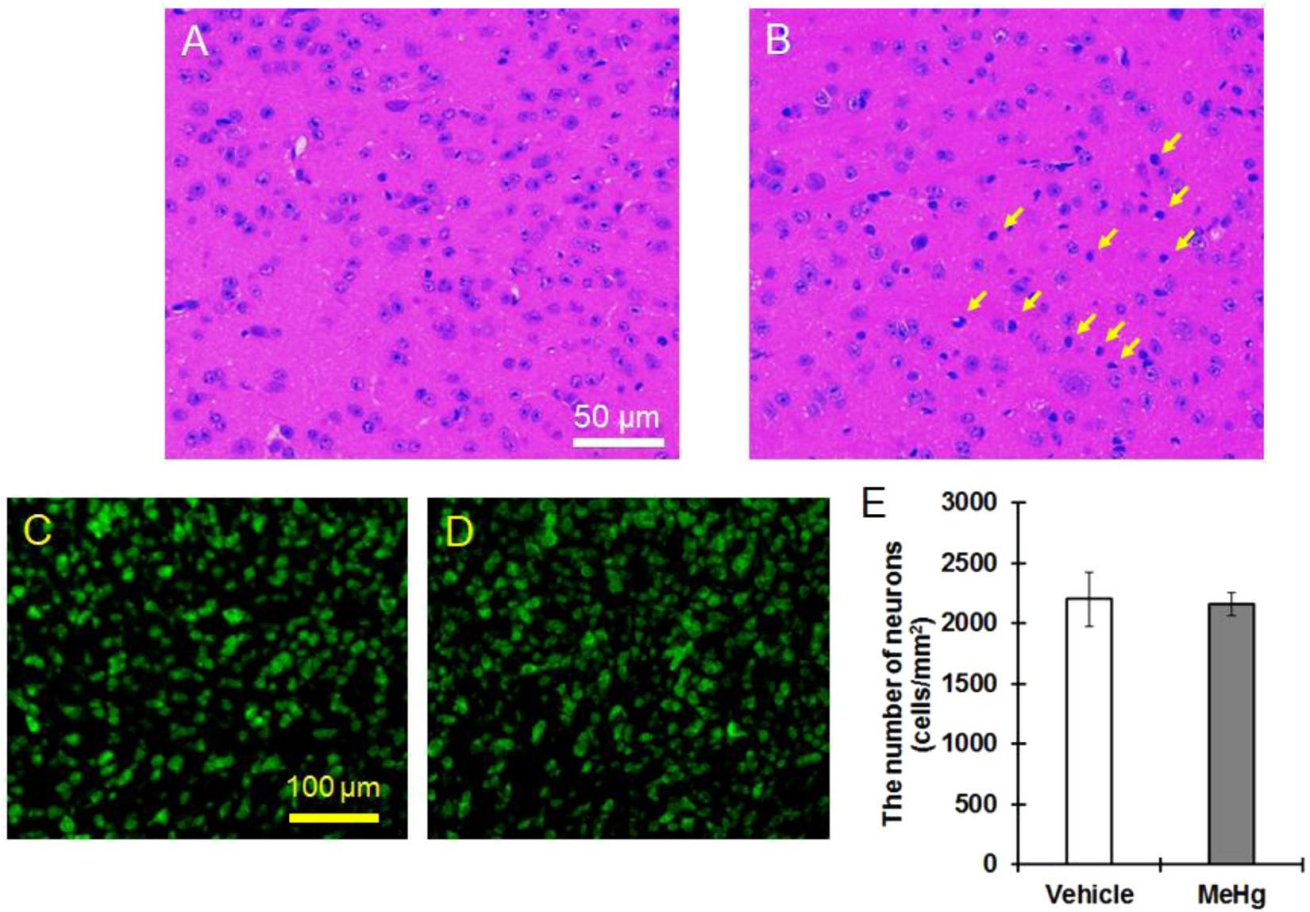
No neuronal loss in the MeHg-exposed IC. MeHg was administered to mice at a dose of 4 mg/kg/day for 4 weeks. HE staining was performed in the IC region of vehicle (**A**) and MeHg-treated (**B**) mice. Arrows indicate dark neurons. Nissl staining was performed in the IC region of vehicle (**C**) and MeHg-treated (**D**) mice, and the number of neurons was counted (**E**). The reported values are the mean ± S.E. (n = 5 animals in each group).

**Figure 4:**
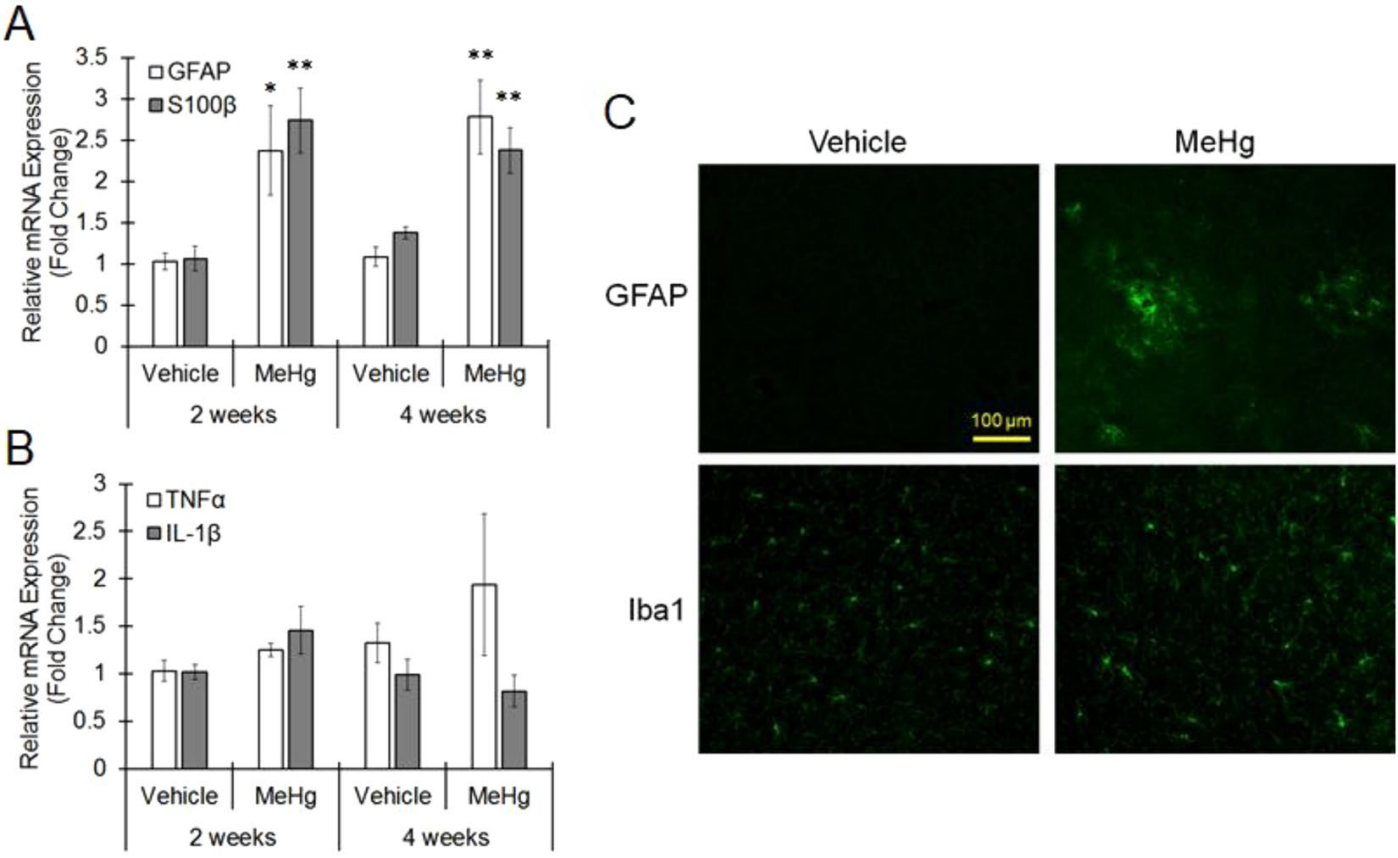
Astrogliosis in the IC induced by MeHg. MeHg was administered to mice at a dose of 4 mg/kg/day for 4 weeks. (**A**,**B**) Total RNA was extracted from the IC region of vehicle and MeHg-treated mice. Real-time PCR was performed to quantify mRNAs for astrocytic markers, GFAP and S100β and for neuroinflammation, TNFα and IL-1β. The reported values are the mean ± S.E. (n = 4 animals in each group). Data were analyzed using a one-way ANOVA, followed by Student’s t test. **P < 0.01 vs. vehicle group with the same age. (**C**,**D**) Frozen sections were prepared from the mice treated with MeHg for 4 weeks and then stained with GFAP (astrocytes) and Iba1 (microglia). The results are representative of 3 independent experiments.

Next, we analyzed the pathology of the IC of MeHg-administered mice. Dark neurons were observed in the IC of mice exposed to MeHg for 4 weeks (indicated by arrows), as evaluated by hematoxylin and eosin (HE) staining (Fig. 3A,B). Based on Nissl staining, there was no difference in the number of neurons between the vehicle and MeHg treatment groups (Fig. 3C–E). The mRNA expression of GFAP and S100β in the IC was significantly increased at 2 weeks and 4 weeks in MeHg-treated mice compared with that in the vehicle-treated mice (Fig. 4A). In the IC region of MeHg-treated mice, a cluster-like structure of GFAP-positive astrocytes was observed (Fig. 4C). On the other hand, MeHg did not affect the expression of TNFα and IL-1β or microglial morphology as evaluated by staining with the microglial marker Iba1 (Fig. 4B,C). These results indicate that astrocyte activation, namely, astrogliosis, occurs in the IC of mice exposed to MeHg.

### Protection of IC neurons from MeHg by activated astrocytes

Increasing evidence shows the diversity of astrocytic activation, such as neurotoxic reactive astrocytes^16^ and phagocytic astrocytes^17^, in addition to a scar^18^, which fills damaged tissue, as reported for a long time. Thus, we next investigated the role of astrogliosis activated by MeHg using an astrocytic activity inhibitor, fluorocitrate (FC)^19^.

**Figure 5:**
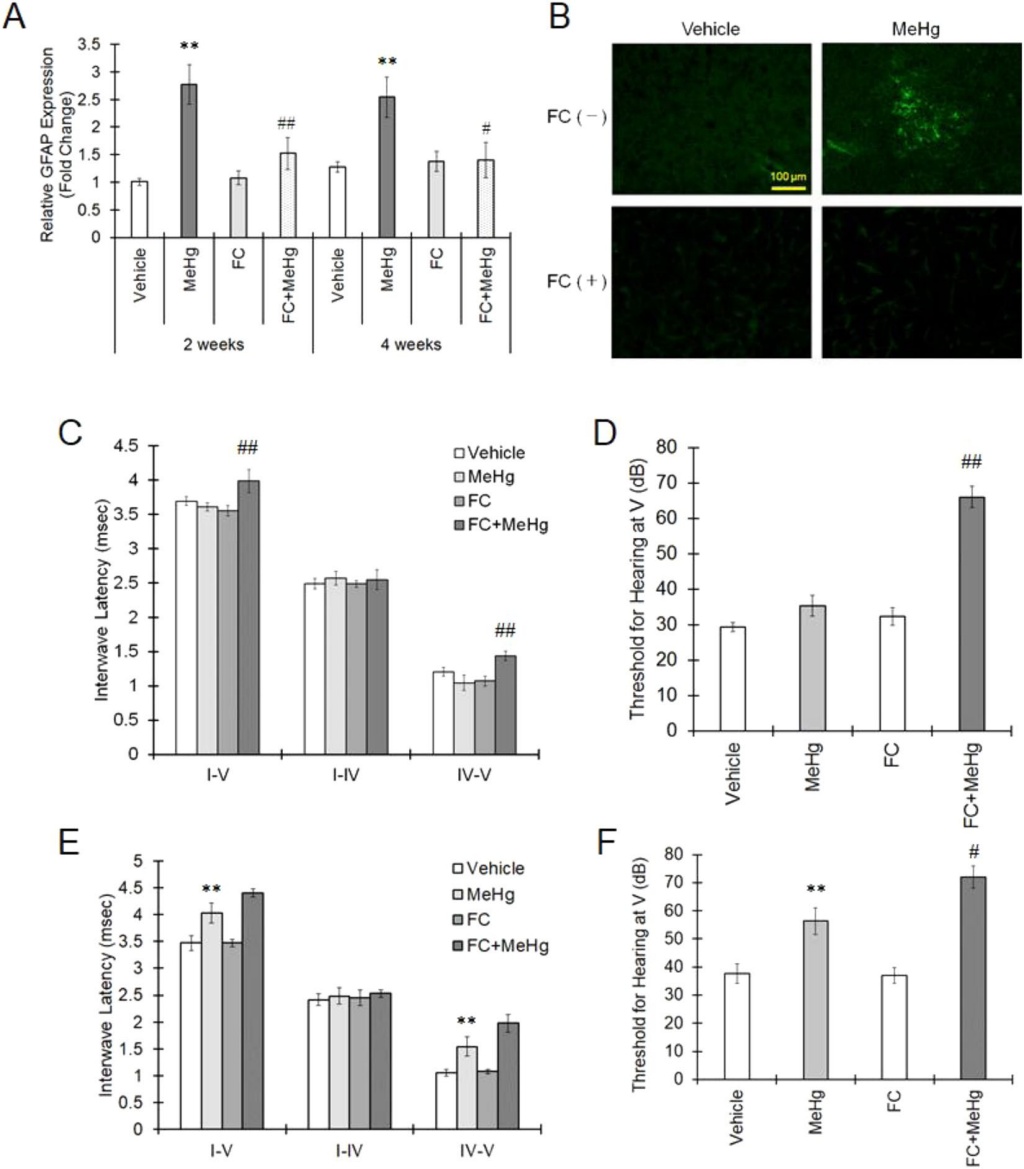
Suppression of astrogliosis and exacerbation of auditory dysfunction by treatment with FC. MeHg was administered to mice at a dose of 4 mg/kg/day for 4 weeks together with 0.3 nmol FC treatment into the lateral ventricle once a week. (**A**) GFAP mRNA expression was assessed by real-time PCR 2 or 4 weeks after the beginning of MeHg administration. The reported values are the mean ± S.E. (n = 5 animals in each group). Data were analyzed using a one-way ANOVA, followed by Student’s t test. Multiple comparisons were assessed by Holm’s corrections. **P < 0.01 vs. vehicle group with the same age. ^#^P < 0.05, ^##^P < 0.01 vs. MeHg group with the same age. (**B**) GFAP expression in the IC region 4 weeks after MeHg administration was evaluated by immunohistochemistry. (C to F) Auditory function 2 weeks (**C**,**D**) or 4 weeks (**E**,**F**) after MeHg treatment was evaluated by ABR measurement. (**C**,**E**) The interwave latency from wave I to wave V, from wave I to wave IV and from IV to V in MeHg-treated mice was measured. The reported values are the mean ± S.E. (n = 5 animals in each group). Data were analyzed using a one-way ANOVA, followed by Student’s t test. Multiple comparisons were assessed by Holm’s corrections. **P < 0.01 vs. the vehicle group. ^##^P < 0.01 vs. the MeHg group. (**D**,**F**) Mice were simulated by different sound pressures with a monitoring evoked potential at wave V to calculate the hearing threshold. Data were analyzed using a one-way ANOVA, followed by Student’s t test. Multiple comparisons were assessed by Holm’s corrections. **P < 0.01 vs. the vehicle group. ^#^P < 0.05, ^##^P < 0.01 vs. the MeHg group.

FC was administered into the lateral ventricle once a week to examine astrocytic activity. The expression of GFAP mRNA, which is increased by methylmercury, is suppressed by FC administration (Fig. 5A). In addition, clusters of GFAP high-expression cells observed in the IC of MeHg-treated mice also disappeared as a result of FC administration (Fig. 5B). These data clearly indicate that FC suppresses astrogliosis in the IC, caused by MeHg exposure. Treatment with FC delayed the evoked potential and increased the threshold at the IC 2 weeks after MeHg administration (Fig. 5C,D). Considering that MeHg administration showed no effect on ABR 2 weeks after exposure but prolonged latency and aggravated the threshold 4 weeks after exposure. Therefore, FC is considered to accelerate hearing loss induced by MeHg. Four weeks after MeHg administration, FC treatment tended to delay the evoked potential elicited by MeHg and significantly exacerbated the increased threshold at the IC induced by MeHg (Fig. 5E,F). In sum, astrogliosis induced by MeHg in the IC has a protective role against IC injury elicited by MeHg.

**Figure 6:**
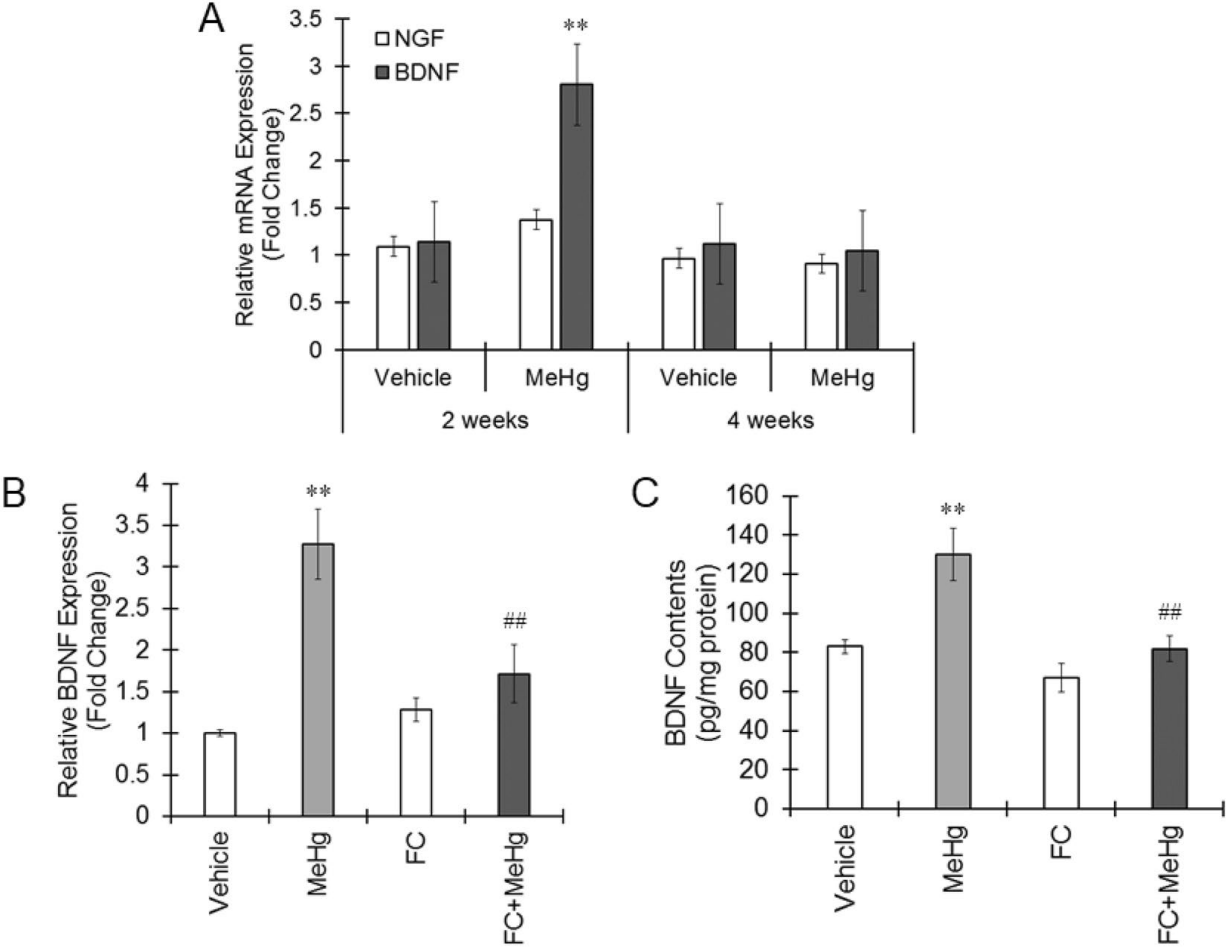
Astrocytic BDNF expression increased by MeHg. (**A**) MeHg was administered to mice at a dose of 4 mg/kg/day for 4 weeks. The expression of NGF and BDNF in the IC region was measured by real-time PCR. Data were analyzed using a one-way ANOVA, followed by Student’s t test. **P < 0.01 vs. vehicle group with the same age. (**B**,**C**) MeHg was administered to mice at a dose of 4 mg/kg/day for 2 weeks together with 0.3 nmol FC treatment into the lateral ventricle once a week. mRNA (**B**) and protein (**C**) expression of BDNF in the IC region was measured by real-time PCR or ELISA, respectively. Data were analyzed using a one-way ANOVA, followed by Student’s t test. Multiple comparisons were assessed by Holm’s corrections. **P < 0.01 vs. the vehicle group. ^##^P < 0.01 vs. the MeHg group.

We have previously reported that primary astrocytes exposed to MeHg protect neurons by synthesizing and releasing BDNF and NGF^11^. BDNF was reportedly expressed in the mouse brain by MeHg exposure^20^. Therefore, we focused on BDNF and NGF as a protectant produced by astrocytes. The expression of BDNF mRNA and proteins was elevated 2 weeks after MeHg exposure but reduced to vehicle levels 4 weeks after MeHg exposure (Fig. 6A,C). There was no change in NGF mRNA throughout the experimental period (Fig. 6A). Increased expression of BDNF mRNA and proteins by MeHg clearly decreased by treatment with FC (Fig. 6B,C). These results indicate that astrocytic BDNF synthesis is potentiated in the IC of mice exposed to MeHg.

## Discussion

Auditory stimulation is detected by the cochlea hair cell and transmitted from the cochlear nucleus via the midbrain to the auditory area of the cerebral cortex. The IC is the auditory nucleus in the midbrain, and all ascending auditory information transmits through the IC to the thalamus. In the IC, complex information processing, such as frequency discrimination of sound, pitch of the sound, speech language, and auditory space recognition, is considered to be performed^21,22^. Pathological analysis of auditory pathways in patients with Minamata disease has been conducted; all patients examined had gliosis, and 55% of the patients showed neurological loss in the IC^13^. Interestingly, in the other nucleus of the auditory pathway, the superior olivary nucleus, 62% of patients showed gliosis, and 23% of patients showed neuronal loss; furthermore, in the lateral lemniscus, 25% of patients showed gliosis, and 8% of patients showed neuronal loss, indicating that these regions can be less sensitive to MeHg than the IC^13^. In addition, all patients with Minamata disease showed gliosis and neuronal loss in the transverse temporal gyrus, which is considered to be extremely sensitive to MeHg^13^. As a mechanism of the occurrence of the specific injury caused by MeHg near the transverse temporal gyrus, the “edema hypothesis” proposed by Eto *et al*. is influential. In this hypothesis, the specificity of the MeHg toxicity in the transverse temporal gyrus is described to be the result of circulatory failure with edema formation in the deep brain groove as an initial lesion^23^. In this study, no significant increases in T2WI and DWI signals were observed until 4 weeks after MeHg administration in any brain region (data not shown), suggesting that IC injury can be induced by mechanisms other than edema. Epidemiological studies of families working in gold mines or people living in the Faroe Islands who ingest a large amount of seafood have shown that mercury exposure affects IC function through the ABR^14,24^. Furthermore, MeHg exposure in mice has been reported to cause delayed conduction and increases in the threshold at the IC region^15^. It is well known that MeHg exposure is equally distributed in the whole brain^25^. In summary, although the mechanism is unclear, the IC is considered to be an early target of MeHg.

In the present study, no neuronal loss was observed at 4 weeks after MeHg exposure when IC dysfunction was detected. At 4 weeks, dark neurons were noted in the IC region by HE staining. Dark neurons have plagued the interpretation of brain sections in both experimental and clinical materials^26^. Dark neurons appear under conditions such as ischemia, epilepsy and Na^+^-K^+^ ATPase inhibition^27–29^. Electron microscopic analysis showed that the membrane and nucleus of dark neurons are intact^30^, suggesting that these cells are still not dead neurons. Hypoglycemia acutely induces the formation of dark neurons, which can go onto recover over several hours^26^, also suggesting the reversibility of dark neuron formation. The dark neuron might be considered a neuron at a risk of undergoing subsequent cell death, but recovery and volumetric expansion may occur en masse in dark neurons. It is unclear whether dark neurons show decreasing or rare function, but IC dysfunction might depend on the formation of dark neurons. As described above, 55% of the Minamata disease patients examined showed neurological loss in the IC^13^. When mice continue to be exposed to MeHg for more than 6 weeks, neurons in the IC decrease, and the IC exhibits a vacuolar degeneration-like histology (data not shown), which is similar phenomena in the Minamata disease. Therefore, the dark neurons observed in this study might have been destined to eventually die.

One of the main mechanisms of MeHg-induced neurotoxicity is oxidative stress. We have reported that mitochondrial ROS injures neurons under MeHg exposure^6^. Because treatment with an antioxidant, namely, vitamin E, at the same time as MeHg administration suppressed the delay of latency and increases in the threshold at the IC (data not shown), oxidative stress generated by MeHg exposure could induce IC dysfunction. The Lin-Shiau research group reported that MeHg exposure decreases the activity of Na^+^/K^+^-ATPase in the brainstem^15,25^. Oxidative stress is suggested to inactivate Na^+^/K^+^-ATPase via lipid peroxidation^31^, and this finding is supported by a report that SOD can recover Na^+^/K^+^-ATPase activity^32^. Therefore, decreases in Na^+^/K^+^-ATPase activity induced by oxidative stress might be a mechanism by which MeHg injured the IC region.

Because astrocytes highly express L-type Amino Acid Transporter 1, which is a transporter for the MeHg-cysteine complex, MeHg accumulates in astrocytes^33,34^. MeHg can induce excitotoxicity via the inhibition of astrocytic glutamate uptake^35^. Astrocytes exposed to MeHg show increased expression of aquaporin-4, resulting in edema formation^36^. In addition, Liddelow *et al*. recently reported that A1 astrocytes activated by inflammatory molecules such as IL-1, TNF and C1q cannot promote neuronal survival but can induce neuronal and oligodendrocytic death^16^. These reports clearly indicate that activated astrocytes have a harmful effect on neurons. On the other hand, astrocytes are known to elicit adaptive reactions responding to neurotoxins to protect neurons^37^. Noguchi *et al*. showed that astrocytes increase IL-6 expression downstream of the ATP-P2Y1 pathway and that IL-6 acts on neurons to reduce MeHg toxicity^38^. We reported that MeHg treatment increased the expression of NGF and BDNF in rat primary astrocytes, which led to neuroprotection from MeHg^11^. In this study, FC treatment suppressed IC dysfunction induced by MeHg, suggesting a protective role of activated astrocytes for neurons. Because BDNF expression was elevated in astrocytes exposed to MeHg for 2 weeks, astrocytes in the IC exhibited adaptive responses by sensing MeHg to suppress neurotoxicity due to BDNF upregulation. However, BDNF expression regulation by MeHg is controversial since BDNF is reportedly downregulated by MeHg^39^. Further experiments are needed to reveal a role of astrocytic BDNF in neuroprotection.

Microglia are more sensitive to endogenous substrates as well as xenobiotics and thus can be the first line of defense against MeHg toxicity. MeHg is reported to activate microglia^12^, but in this study, microglia in the IC were not activated, although TNFα expression tended to be increased by MeHg. MeHg showed a significant increase in ROS production in microglia only 1 min after exposure when astrocytes were not activated^40^. Therefore, microglia might be activated 1 week or less after MeHg exposure. Microglia activated by MeHg can be a trigger to astrocyte-mediated neuroprotection^12^, and astrocytes are converted to a cytotoxic form by activated microglia^16^. These findings suggest that astrocytic function could be affected by the degree and/or duration of microglial activation. The astrocyte-microglia interaction in the auditory pathway should be investigated in the future.

## Conclusion

MeHg induced IC dysfunction before it caused a coordinated movement disorder. Activation of the astrocytes occurred in the IC exposed to MeHg, and inhibition of astrocytic activation accelerated the damage to the IC, indicating that astrocytes activated in the IC play a protective role for neurons. Auditory disorder induced by MeHg could be determined by the balance between the damage to neurons by MeHg and the protective action of astrocytes activated by MeHg. Therefore, elucidation of cell-cell interactions might be important for understanding the mechanism of MeHg toxicity.

## Materials and Methods

### Animals

All animal procedures were performed in accordance with the Fundamental Guidelines for Proper Conduct of Animal Experiments and Related Activities in Academic Research Institutions under the jurisdiction of the Ministry of Education, Culture, Sports, Science and Technology, Japan. The Animal Care and Use Committee of Hiroshima University approved the experimental protocols. Male ICR mice were obtained from Japan SLC (Shizuoka, Japan) and were maintained in a temperature-controlled animal facility with a 12-h light-dark cycle.

### Animal treatment

Methylmercury chloride (Tokyo Chemical Industry, Tokyo, Japan) was dissolved in Dulbecco’s phosphate-buffered saline (PBS), including an equal amount of L-cysteine and was orally administered at a dose of 4 mg/kg every day. Fluorocitrate (FC) solution for intrastriatal injection was prepared according to the previous report^41^. Eight milligrams of dl-fluorocitric acid barium salt (Sigma-Aldrich, St. Louis, MO, USA) was dissolved in 1 ml of 0.1 mmol/L HCl. Two to three drops of 0.1 mmol/L Na_2_SO_4_ were added to precipitate the barium ion. Two milliliters of 0.1 mmol/L Na_2_HPO_4_ was added, and then the suspension was centrifuged at 1,000 *g* for 5 min. The supernatant was diluted with PBS and used for treatment. FC was administered into the lateral ventricle at 0.3 nmol/5 μL/mouse once a week.

### Rotor rod test

A rotarod apparatus (LE8200, Panlab, Barcelona, Spain) was used to test the ability of mice to coordinate movements. The rotarod test was performed by placing a mouse on a rotating drum and measuring the time each animal was able to maintain its balance walking on top of the rod. The speed of the rotarod accelerated from 4 to 40 rpm over a 5-min period. Mice were given 2 trials with a maximum time of 5 min and a 60 min intertrial rest interval. The average latency from 2 trials was calculated.

### MRI

We obtained coronal MR images in all mice using T1WI, T2WI, and DWI sequences, in accordance with our previous report^42^. Briefly, mice were anesthetized with isoflurane, and body temperature was maintained at a constant 37 ± 0.2 °C. The MRI data were acquired using an MRmini-SA (DS Pharma Biomedical, Osaka, Japan), consisting of a 1.5-Tesla permanent magnet, a compact computer-controlled console, and a solenoid MRI coil with a 30-mm inner diameter. We obtained coronal MR images using a 2D spin-echo (SE) multislice (MS) T1WI sequence, FOV = 20 × 40 mm^2^, matrix = 128 × 256, voxel size = 0.234 × 0.234 × 1.0 mm and a slice thickness of 1.0 mm for all 11 contiguous coronal MR images. The typical T1WI parameters were TR (ms)/TE (ms) = 500/9, NEX = 8. After T1WI was performed, T2WI was conducted. T2-weighted images and diffusion-weighted images were obtained with the following parameters: the T2 WI parameters were TR (ms)/TE (ms) = 2500/69, NEX = 4, and the DWI parameters were TR (ms)/TE (ms) = 2500/69, b value = 850 sec/mm^2^, NEX = 4. The images were analyzed using an INTAGE Realia Professional software program (Cybernet Systems Co., Ltd., Tokyo, Japan) and ImageJ software (National Institutes of Health, Bethesda, MD, USA).

### ABR

Auditory function of the mice was evaluated by the system for ABR (Tucker-Davis Technologies, Alachua, FL, USA)^43^. Mice were anesthetized with pentobarbital (50 mg/kg i.p.), and their body temperature was maintained at 36–37 °C. Brainstem-evoked responses to sound stimuli were recorded by subdermal needle electrodes, which were placed on the vertex and ipsilateral retroauricular region. A ground electrode was placed on the backs of the mice. ABR responses were elicited by clicks at increasing sound intensities ranging from 0 to 90 dB sound pressure levels in 5 dB steps, and the obtained evoked potentials were averaged 500 times. Latency between peaks and thresholds, the lowest sound level that evoked a visible ABR, were determined.

### HE and Nissl staining

For the histological analyses, mice were deeply anesthetized and euthanized with sodium pentobarbital (50 mg/kg, Sigma-Aldrich) and perfused transcardially with heparinized 0.1 M PBS, followed by 4% paraformaldehyde (PFA) in 0.1 M PBS, pH 7.4. After perfusion, the brains were removed and postfixed overnight in 4% buffered PFA at 4 °C and then cryoprotected in 30% sucrose. Paraffin sections were prepared, followed by HE staining. Serial frozen sections (10 μm) were cut on a sliding Cryostat (CM3050S, Leica Biosystems, Nussloch, Germany), mounted onto slides, and then the sections were stained with NeuroTrace 500/525 Green Fluorescent Nissl Stain (Thermo Fisher Scientific, Waltham, MA, USA) according to the manufacturers’ protocol.

### Immunohistochemistry

GFAP and Iba1 staining was performed according to our previous report^44^. The brains were removed, postfixed overnight in 4% buffered PFA at 4 °C after perfusion and cryoprotected in 30% sucrose. Brains were frozen in ethanol with dry ice, and 10-μm-thick sections were prepared using a Cryostat. The sections were blocked and permeabilized with PBS including 10% normal goat serum (Sigma-Aldrich) and 0.3% Triton-X 100 for 1 h at room temperature. The sections were incubated with primary antibody (Anti-GFAP, no dilution, Dako/Agilent, Santa Clara, CA, USA; Anti-Iba1, 1/500, Wako, Osaka, Japan) for 3 h at room temperature, followed by Alexa-labeled secondary antibody for 1 h at room temperature in the dark. The sections were mounted on a glass slide with DAPI-Fluoromount-G (Southern Biotech, Birmingham, AL, USA). Images were obtained with an inverted fluorescence microscope (BZ-9000, Keyence, Osaka, Japan) and were processed using ImageJ software (NIH, MD, USA).

### Total RNA extraction and real-time PCR

mRNA levels were determined by methods previously reported^45^. Briefly, total RNA was extracted from microglia using a High Pure RNA Isolation Kit (Roche Diagnostics K.K., Tokyo, Japan). Single-stranded cDNA was synthesized from 1 μg of total RNA according to the ReverTra Ace protocol (Toyobo, Osaka, Japan) with a random primer (9-mer; Takara Bio, Ohtsu, Japan). Real-time PCR was performed using a CFX Connect instrument (Bio-Rad, Hercules, CA, USA) with TB Green Premix Ex Taq II (TaKaRa). The primer sequences used in this study are listed in Table 1. mRNA levels were corrected to levels of β-actin mRNA, and relative mRNA levels were calculated by dividing treated mice levels by control mice levels.

**Table 1:** Primer sequences used for real-time PCR.

Target	Forward primer (5′-3′)	Reverse primer (5′-3′)
Mouse GFAP	AGGGCGAAGAAAACCGCATC	GGTGAGCCTGTATTGGGACA
Mouse S100β	TTCCTGGAGGAAATCAAGGAGC	GGAAGTCACACTCCCCATCC
Mouse TNFα	ATGGCCTCCCTCTCATCAGT	CTTGGTGGTTTGCTACGACG
Mouse IL-1β	AGCTTCCTTGTGCAAGTGTCT	GCAGCCCTTCATCTTTTGGG
Mouse NGF	TCATCCGGATAGACACAGCC	ACAGGCTGAGGTAGGGAGG
Mouse BDNF	CCGGTATCCAAAGGCCAACT	CTGCAGCCTTCCTTGGTGTA
Mouse β-actin	CTAGGCACCAGGGTGTGATG	GGGGTACTTCAGGGTCAGGA

### Determination of BDNF contents

The levels of BDNF present in the mouse brain were evaluated with the BDNF Emax ImmunoAssay System (Promega, Madison, WI, USA) according to the manufacturer’s instructions^11^.

### Statistical analyses

All of the data are expressed as the mean ± standard error (S.E.). The statistical analyses were performed using a one-way analysis of variance (ANOVA), followed by Student’s t test. Multiple comparisons were assessed by Holm’s corrections. P values of < 0.05 were considered to indicate statistical significance.

## Data Availability

The datasets generated during and/or analyzed during the current study are available from the corresponding author on reasonable request.
